# A critical appraisal of perioperative sleep apnoea management after nasal surgery: A review of up-to-date literature supplemented by findings of a retrospective observational study

**DOI:** 10.1177/17504589231215941

**Published:** 2024-01-11

**Authors:** Anne Duvekot, Markus Klimek, Frank R Datema

**Affiliations:** 1 Department of Otorhinolaryngology, Head and Neck Surgery, Erasmus University Medical Center, Rotterdam, The Netherlands; 2 Department of Anesthesiology, Erasmus University Medical Center, Rotterdam, The Netherlands

**Keywords:** Obstructive sleep apnoea, Post-anaesthesia care unit, Nasal surgery, Respiratory events

## Abstract

**Objective:**

To review the current recommendations on postoperative precautions for obstructive sleep apnoea patients undergoing elective nasal surgery.

**Design:**

Retrospective cohort study.

**Setting:**

Department of Otorhinolaryngology and Anesthesiology/Intensive Care, University Teaching Hospital, Rotterdam, the Netherlands.

**Participants:**

The medical charts of 61 patients with sleep apnoea who were admitted to the post-anaesthesia care unit between 2016 and 2020, following nasal surgery were reviewed.

**Main outcome measures:**

Number of respiratory events during post-anaesthesia care unit admission that required medical intervention.

**Results:**

In all 61 patients, continuous positive airway pressure could not be used. In 13 patients (8%), decreased oxygen saturation levels were registered during the first postoperative night, and in five of these patients, supplemental oxygen was needed. No other respiratory incidents of medical interventions were registered.

**Conclusions:**

The number of clinically relevant respiratory events of obstructive sleep apnoea patients with bilateral nasal packing following nasal surgery is low. We suggest that the safety of less expensive and less scarce alternatives of postoperative observation should be explored.

## Introduction

Obstructive sleep apnoea (OSA) is associated with an increased risk for perioperative neurological, pulmonary and cardiovascular complications (Zaremba et al 2016). This risk increases when opioids are used because OSA patients need higher doses and are more sensitive to breathing suppressions (Chung et al 2014). Severe perioperative complications may result in intensive care unit (ICU) admission or a prolonged hospital stay. National OSA care guidelines and local preoperative OSA screening protocols are mainly directed at the prevention of these scenarios (ASA, Mokhlesi et al 2013, Dutch Society of Otolaryngology/Head-Neck Surgery (NVKNO) Dutch Society of Pulmonology and Tuberculosis (NVALT) 2018, Zaremba et al 2016). In this article, we investigate if the gain of preventive admission at the post-anaesthesia care unit (PACU) is justified in OSA patients who could not use continuous positive airway pressure (CPAP) because of occlusive nasal packing.

In the Erasmus Medical Center, every diagnosed OSA patient who cannot use CPAP following surgery is admitted to the PACU. The PACU is best compared to a medium-level ICU, where patients are monitored with continuous pulse oximetry and electrocardiography. In addition, every patient with an STOP-BANG score of ⩾5 is considered to be at risk of OSA. The STOP-BANG questionnaire is a routine part of the preoperative anaesthesia screening protocol to identify OSA risk factors and has been validated in surgical and sleep clinic settings worldwide (Chen et al 2021). In case of elective surgery, there is time to offer these patients polysomnography (PSG). When PSG confirms OSA, these patients are admitted to the PACU as well. After one night of uneventful observation, patients are transferred to a general unmonitored ward or discharged home. When prolonged monitored observation is indicated, patients are transferred to the ICU.

Bilateral occlusive nasal packing is common after septoplasty, septorhinoplasty, or functional endoscopic sinus surgery (FESS). Subsequently, CPAP-dependent OSA patients cannot wear a nasal or full-face mask and, according to our local protocol, are admitted to the PACU. However, the PACU has a very limited capacity, which impacts surgical planning and waiting lists. Furthermore, PACU admission is more expensive (1,700 EUR/night) than admission on a general ward (470–890 EUR/night). More importantly, the literature reports few respiratory and other complications in the early postoperative phase of the abovementioned patient group (Friedman et al 2011, Regli et al 2006). Therefore, protocolled PACU admission might be redundant.

Especially in times of scarce resources, long waiting lists and increasing health care costs, safe but less expensive alternatives might be indicated and should be explored. This retrospective study investigates the gain (clinically relevant respiratory findings requiring interventions to avoid perioperative complications) of PACU admittance in OSA patients with occlusive nasal packing.

## Methods

### Patient population and study parameters

The medical charts of all OSA patients who were admitted to the PACU following septoplasty, septorhinoplasty or functional endoscopic sinus surgery (FESS) between 2016 and 2020 were collected. Patients with comorbidities other than OSA, forming another strict PACU admission were excluded from analysis. The medical charts of the remaining patients were reviewed for demographic characteristics, STOP-BANG score, OSA severity, preoperative OSA treatment (CPAP, mandibular repositioning device (MRA)), type of nasal packing, postoperative respiratory events and management, postoperative use of opioids and use of supplemental oxygen.

All data were stored in an SPSS database for further analysis.

## Results

### Study population

Between 2016 and 2020, 155 consecutive OSA patients were admitted to the PACU following nasal surgery (Tables 1 and 2). Eighty-six patients were excluded from analysis because they did not require occlusive nasal packing. Eight more patients were excluded because other comorbidity formed a strict indication for postoperative PACU admission. The final study population consisted of 61 OSA patients who received bilateral nasal packing following nasal surgery. The mean age of the population was 53.9 (range = 23–75) years and the majority were males (60.7%). Forty patients (65.6%) underwent septoplasty or septorhinoplasty. Transsphenoidal pituitary gland surgery was performed in seven patients (11.5%) and FESS in 14 patients (22.9%). From the 61 patients, 59 (96.7%) patients were preoperatively known with OSA, of which 39 (63.9%) used CPAP and three (4.9%) an MRA device. Twenty patients were known with OSA but received no treatment. Two undiagnosed OSA patients were pragmatically admitted to the PACU because of a high STOP BANG score. In contrast to protocol and for unknown reasons, they did not receive a preoperative PSG.

**Table 1:** Patient characteristics

	n (% of total 61 patients)
Gender	Male 37 (60.7%)
Age, median (range)	54 (23–75)
Performed surgery	
(Septo)rhinoplasty	40 (65.6%)
FESS (including one endoscopic hypophysectomy)	14 (23.0%)
Transseptal hypophysectomy	7 (11.5%)
Postoperative nasal packing	
Bilateral	52 (85.2%)
Unilateral	7 (11.5%)
Ethmoidal	2 (3.2%)
Way of preoperative OSA diagnosis	
None (already known)	59 (96.7%)
STOP-BANG	2 (3.3%)
PSG	0
Perioperative opioid use	24 (39.3%)
Preoperative OSA treatment	
CPAP	39 (63.9%)
MRA	3 (4.9%)
Other (oxygen)	1 (1.6%)
None	18 (29.5%)
Postoperative PACU admission according to protocol	
YES	58 (95.1%)
NO	3 (4.9%)
Respiratory events during PACU admission	
Apnoeas without decreased oxygen saturation levels	1 (1.6%)
Apnoeas with decreased oxygen saturation levels	2 (3.3%)
Supplemental oxygen administration	0 (0.0%)
Decreased oxygen saturation levels without apnoeas	11 (18.0%)
Supplemental oxygen administration	4 (36.4%)^a^
None	47 (77.0%)
With extra oxygen administration	1 (2.1%)^b^

FESS: functional endoscopic sinus surgery; OSA: obstructive sleep apnoea; PSG: polysomnography; CPAP: continuous positive airway pressure; MRA: mandibular repositioning device; PACU: post-anaesthesia care unit.

a Four of 11 equals 36.4%.

b One of 47 equals 2.1%; this patient received extra oxygen administration on personal (comforting) request.

**Table 2:** Indications for postoperative admittance at the PACU for OSA patients at the Erasmus Medical Center Rotterdam (columns in blue). Admittance numbers to the PACU from our study population (column in grey).

	PACU	Unmonitored setting	PACU (%)
			Total N = 61
OSAS with use of own device		X	2 (3.3%)
OSAS, own device can temporarily not be used, no need for postoperative opioids	X		18^a^ (29.5%)
OSAS, own device can temporarily not be used, need for postoperative opioids	X		38 (62.3%)
STOP-BANG ⩾ 5, use of short-acting and/or local anaesthesia, no need for postoperative opioids		X (after two hours of observation at the recovery)	1 (1.6%)
STOP-BANG ⩾ 5, need for postoperative opioids (including epidural anaesthesia)	X		2 (3.3%)
STOP-BANG ⩽ 5		X	0 (0.0%)

PACU: post-anaesthesia care unit; OSA: obstructive sleep apnoea; OSAS: obstructive sleep apnoea syndrome.

a Including the previously described patient who went home against medical advice.

### Postoperative observations

Opioids (morphine and/or oxycodone and/or piritramide) were prescribed in 42 patients (68.8%) and effectively administered in 24 patients (39.3%).

Decreased oxygen saturation levels, defined as a 5% saturation drop from baseline or an absolute value of <90%, were observed in 13 patients (21.3%). Seven of these 13 patients (53.8%) received opioids. The lowest saturation level was 80% and present in one patient (who did not receive opioids). Only five of these 13 patients required oxygen supplementation. In two other patients, apnoeas were registered but without decreased saturation levels. No other incidents or interventions were reported. All patients could be transferred to a ward after one night of observation and were discharged home that day.

## Discussion

The reported prevalence of OSA worldwide is highly variable because of different diagnostic definitions and lifestyle-related phenotypes. Independently, the prevalence of OSA is increasing (Farney et al 2011, Heinzer et al 2015, Memtsoudig et al 2014, Zaremba et al 2016). In the Netherlands, the prevalence of OSA is estimated to be 2% to 3% of the general population, but these percentages are presumably higher when patients without daytime symptoms are considered (Dutch Society of Otolaryngology/Head-Neck Surgery (NVKNO) Dutch Society of Pulmonology and Tuberculosis (NVALT) 2018).

Besides the long-term consequences of untreated OSA and its implications on health care consumption in general, OSA patients have an increased risk of respiratory complications in the perioperative phase. Severe complications may result in ICU admission or a prolonged hospital stay. The prevention of such complications is a general responsibility of involved healthcare providers and anesthesiologists specifically. Therefore, the liberal use of preventive perioperative management is understandable, especially in case of elective surgical procedures. Unfortunately, randomised controlled trials to scientifically substantiate specific precautions in perioperative OSA care are lacking, and most recommendations are, therefore, based on expert opinion.

In our hospital, independent of OSA severity or at-home OSA treatment, all diagnosed patients, or patients with an increased risk of OSA, according to the STOP-BANG questionnaire, who are unable to receive CPAP after surgery, are admitted to the PACU for at least one postoperative night of observation. Subsequently, a relatively high PACU admission rate occurs in our surgical rhinology population that receives occlusive nasal packing.

In times of scarce resources and increasing healthcare costs, we felt the responsibility to investigate the gain of postoperative PACU admission in this specific patient group. We found respiratory depressions in 13 of 61 patients, with only one case of severe desaturation to 80%. Five patients received supplemental oxygen and no other noteworthy events or interventions were encountered. These data illustrate a limited gain of PACU admission and form a sufficient basis to critically appraise local preventive OSA management and to explore more cost-effective and less scarce alternatives to monitor these patients.

In hindsight, the decision for PACU admittance seems too liberal or too pragmatic. This is illustrated by the fact that of 19 diagnosed OSA patients, there was no information about their at-home OSA treatment. Patients, for example, who are successfully managed by an MRA device, could have continued wearing the device after surgery even in cases with occlusive nasal packing. Furthermore, PACU admission of the two at-risk patients with a high STOP-BANG score might have been avoided if time was taken to perform polysomnography. On the contrary, the actual number of clinically relevant OSA patients is potentially underestimated by using an STOP-BANG cut off value of ⩾5. Literature reports an increased OSA risk when the STOP-BANG score is ⩾3 and a rather confident exclusion of moderate to severe OSA in case of a score of 0–2 (Farney et al 2011, Vasu et al 2010).

One postoperative night of PACU admittance might give a false impression of safety. Literature reports the greatest risks of respiratory events during rapid eye movement (REM) sleep at postoperative day three to four, when regular sleep patterns are reestablished (ASA 2014, Mokhlesi et al 2013). Since nasal packing remains in place until day two to four after surgery, it is fair to assume that most respiratory events will occur at home. Another factor that might limit the true preventive value of PACU admittance is that patients report a lack of sleep during the first night after surgery caused by perioperative anxiety, intermittent checks by the nursing staff and noisy alarm bells at the unit. Subsequently, the phase of deep sleep is not easily reached. It has not escaped our attention that this might have influenced the limited number of severe apnoeas and desaturations in this study.

This retrospective observational study is limited mainly due to possible inadequate or incomplete medical chart notations. However, we feel that it can be assumed that great respiratory complications and interventions, other than oxygen supplementation, would have been denoted. Based on previous literature and the outcome of our own study, we suggest a few alterations in perioperative OSA management to be considered.

First of all, as denoted before and according to our local protocol, preoperative OSA screening is performed by the STOP-BANG questionnaire. When STOP-BANG criteria are ⩾5 and in the case of elective surgery, patients are referred to the otorhinolaryngology department for PSG analysis. If the presence of OSA is confirmed, preoperative CPAP is indicated.

However, our anesthesiologists confirmed that in daily practice with long PSG waiting lists and great volumes of oncologic surgery that cannot be postponed, few patients actually undergo further OSA diagnostics before surgery. Sequentially, all patients with STOP-BANG criteria ⩾5 are eligible for postsurgery PACU admission without confirmed diagnosis and without adequate preoperative treatment. Literature reports that in orthopaedic patients with preoperative at-home CPAP use, postoperative respiratory complications are less common compared to a control group (Gupta et al 2001). Since nasal surgery in our hospital is mostly elective surgery, there should be no need to diverge from protocol.

Second, according to our local criteria, OSA patients who are adequately treated with CPAP therapy and able to use their at-home device after surgery, can be observed at an unmonitored ward. Literature shows that when patients are hindered from using their CPAP device due to nasal packing, they can quite easily adjust their CPAP with a mouth piece to temporarily use it as an oral device (Dorn et al 2001). By using this method in our study, postoperative PACU admission could have been averted in 55 patients (90%).

Third of all, as mentioned before, the perioperative risk for OSA patients is further increased by opioid use. Opioid use in the days after surgery, when oximetry monitoring is ceased, can cause unnoticed respiratory events. An unofficial inventory among our local rhinologists and anesthesiologists reveals that the use of postoperative opioids after nasal surgery is often unnecessary. Accordingly, in our study population, we see that despite the prescription in all patients, the actual use of postoperative opioids is only seen in 24 patients (39.3%) and is mostly limited to the day of, or the next morning, after surgery. If necessary, pain management during the following days after surgery should be optimised with non-steroidal anti-inflammatory drugs to reduce opioid use (Zaremba et al 2016).

Finally, and frankly most importantly, in general there is no scientific evidence for postoperative monitoring of OSA patients at a critical care ward. According to literature, it is safe to monitor OSA patients on a general hospital ward with pulse oximetry in the presence of an appropriately trained healthcare professional (ASA 2014).

Based on our results, we suggest a prospective trial where postoperative, PSG-confirmed OSA patients, who are unable to continue the use of CPAP or MRA device, and in the absence of other intensive care needing comorbidities, are monitored by pulse oximetry on a dedicated postoperative OSA hospital ward under the condition that supplemental oxygen administration can be administered when necessary. With positive results to be expected, we hope that this study will support a safe, cost-effective and logistically interesting alternative to current local policy.

## Conclusion

The number of clinically relevant respiratory events of OSA patients with bilateral nasal packing following nasal surgery is low. Although numbers in this study are low, the impact of postoperative opioid use on the occurrence of respiratory events or complications in the first night after surgery seems to be negligible. Therefore, the gain of PACU admittance in these patients is low. The safety of less expensive and less scarce alternatives, such as continuous pulse oximetry on a general ward, should be explored.

## Key points

- Postoperative precautions for OSA (obstructive sleep apnoea) patients undergoing elective nasal surgery.
- Occurrence of postoperative respiratory events in OSA patients admitted to the post-anaesthesia care unit (PACU) with bilateral occlusive nasal packing following septoplasty, septorhinoplasty or sinus surgery.
- Examination of the clinical value of postoperative monitoring at the PACU for OSA patients who needed to temporarily discontinue (continuous positive airway pressure (CPAP)).
- Evaluation of the safety of less expensive and less scarce alternatives than PACU admission for postoperative OSA patients.

## Declarations

**Availability of data and materials:** The data sets used and/or analyzed during the current study are available from the corresponding author on reasonable request.

**Ethical approval:** Ethical approval for the study was not required.

**ORCID iD:** Anne Duvekot  https://orcid.org/0000-0001-5845-6711
